# Adipose Tissue-Derived Mesenchymal Stem Cells Suppress Growth of Huh7 Hepatocellular Carcinoma Cells via Interferon (IFN)-β-Mediated JAK/STAT1 Pathway *in vitro*

**DOI:** 10.7150/ijms.41354

**Published:** 2020-02-18

**Authors:** Chun Sung Byun, Soonjae Hwang, Sung-Hun Woo, Moon Young Kim, Jin Suk Lee, Jong In Lee, Jee Hyun Kong, Keum Seok Bae, Il Hwan Park, Sung Hoon Kim, Young Woo Eom

**Affiliations:** 1 Department of Cardiovascular and Thoracic Surgery, Yonsei University Wonju College of Medicine, Wonju, Gangwon-do 26426, Republic of Korea.; 2 Regeneration Medicine Research Center, Yonsei University Wonju College of Medicine, Wonju, Gangwon-do 26426, Republic of Korea.; 3 Department of General Surgery, Yonsei University Wonju College of Medicine, Wonju, Gangwon-do 26426, Republic of Korea.; 4 Department of Biomedical Laboratory Science, College of Health Sciences, Yonsei University, Wonju, Gangwon-do, 26493, Republic of Korea.; 5 Department of Internal Medicine, Yonsei University Wonju College of Medicine, Gangwon-do 26426, Republic of Korea.; 6 Cell Therapy and Tissue Engineering Center, Yonsei University Wonju College of Medicine, Wonju, Gangwon-do 26426, Republic of Korea.

## Abstract

Interferon (IFN)-β and/or tumor necrosis factor-related apoptosis-inducing ligand (TRAIL) secreted by adipose tissue-derived mesenchymal stem cells (ASCs) have been proposed as key mechanistic factors in anti-cancer efficacy in lung cancer and breast cancer cells, where they act through paracrine signaling. We hypothesized that IFN-β and TRAIL produced by ASCs suppress proliferation of hepatocellular carcinoma cells (HCCs). The present study evaluated the anti-cancer effects of ASCs on HCCs *in vitro*. We found that indirect co-culture with ASCs diminished growth of Huh7 hepatocellular carcinoma cells with increased protein levels of p53/p21 and phosphorylated STAT1 (pSTAT1), without apoptosis. Treatment with ASC-conditioned medium (ASC-CM) also decreased growth of Huh7 cells through elevated p53/p21 and pSTAT1 signaling. ASC-CM-mediated inhibition of cell growth was neutralized in Huh7 cells treated with anti-IFN-β antibody compared to that in ASC-CM-treated Huh7 cells incubated with an anti-TRAIL antibody. Treatment with JAK1/JAK2 inhibitors recovered inhibition of growth in Huh7 cells incubated in ASC-CM or IFN-β via down-regulation of pSTAT1/p53/p21. However, treatment of IFN-β resulted in no alterations in resistance of Huh7 cells to TRAIL. Our findings suggest that ASCs decrease growth through activated STAT1-mediated p53/p21 by IFN-β, but not TRAIL, in Huh7 cells.

## Introduction

Hepatocellular carcinoma (HCC) is the second most common cause of cancer-related deaths globally 1-2. Most HCC patients present at an advanced stage of disease, making them ineligible for curative surgery. Therapeutic options are limited in their availability and efficacy, with the multi-kinase inhibitor Sorafenib only extending survival by 3 months 3-4. Therefore, there is an urgent need for new HCC treatments.

Mesenchymal stem cells (MSCs) are bone marrow-derived cells that can differentiate into mesodermal cell lineages, and are easily isolated and propagated *in vitro* 5. MSCs can differentiate into a number of mesodermal cell lineages, including bone, cartilage, stroma, adipose, connective tissue, muscle and tendon 6. Therefore, MSCs that maintain their capacity for self-renewal ability contribute to a wide variety of endogenous organ and tissue repair 7. In this rapidly expanding field, there is an increased focus on the relationship between stem and tumor cells. Growing evidence demonstrates that MSCs localize to sites of tumorigenesis, where they inhibit tumor cell function, although there is evidence to suggest that MSCs may suppress tumor growth 8-10. Within the tumorigenic environment, MSCs induce neovascularization, up-regulate tumor-promoting stromal networks, induce immune tolerance, and increase metastasis 11-13. In contrast, MSCs are recruited with high tumor specificity to gliomas in the brain, and they prolong the survival of tumor-bearing animals 12-13. MSCs also localize to sites of Kaposi's sarcoma, and potently inhibit tumor growth *in vivo* by downregulating AKT activity in tumor cells cultured with MSCs prior to transplantation in animal tumor models 10.

Mesenchymal stem cells (MSCs) have emerged as ideal agents for the restoration of damaged tissues in clinical applications due to their undifferentiated cell characterization, self-renewal ability with a high proliferative capacity, their paracrine, trophic effect and their mesodermal differentiation potential 11-12. Additionally, they produce bioactive anti-inflammatory agents and support regeneration of injured tissues 13.

Previously, we reported that ASCs, cultured at high-density, suppress tumor growth in MCF-7 breast cancer cells and H460 lung cancer cells through secretion of interferon (IFN)-β or tumor necrosis factor-related apoptosis-inducing ligand (TRAIL), separately 14-15. According to previous studies, ASC-mediated inhibitory effects on tumor growth may depend on the type or origin of cancer cells. However, mouse B16 melanoma cells rapidly develop resistance to the anti-proliferative effects of IFN-β when they are exposed to the interferons *in vitro* 15. TRAIL, a member of the TNF superfamily, is able to induce programmed death in cancer cells with no toxicity against normal tissues 14. However, many studies have demonstrated resistance of most HCC cells to TRAIL-mediated apoptosis 17-22.

Hajighasemlou et al. reported the potential of MSCs to migrate to the tumor microenvironment of HCC in mice with no significant alterations in tumorigenesis, and biochemical markers indicating liver or kidney damage 23. Whilst other study indicates suppression of proliferation and induction of apoptosis in two types of HCC (HepG2 and PLC-PRF-5 cells) indirectly co-cultured with ASCs 24, the anti-tumorigenic mechanism of MSCs and their secretion-mediated effects are not yet clear.

Therefore, the role of MSCs on HCC remains controversial, and few reports have investigated the effects of ASCs on HCC.

Here, we investigated the potential of ASCs to decrease growth of HCCs, and studied mechanisms of ASC-mediated tumor inhibition in terms of IFN-β and/or TRAIL, and evaluated TRAIL sensitivity in the presence of IFN-β in TRAIL-resistant Huh7 hepatocellular carcinoma cells *in vitro*.

## Results

### Morphological and immunological characterization of adipose-derived mesenchymal stem cells

**Figure 1:**
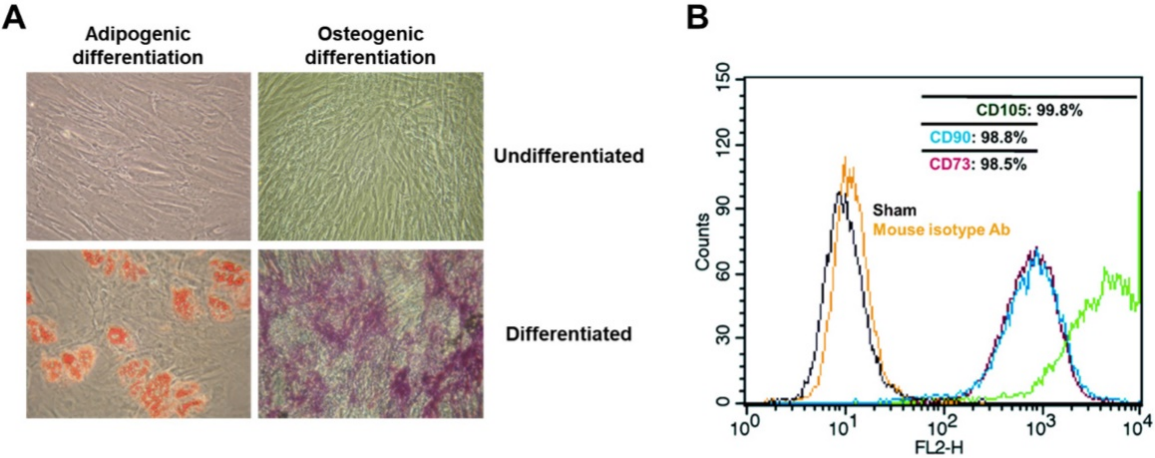
** Morphological and immunological identification of adipose tissue-derived mesenchymal stem cells.** 1 x 10^6^ unstimulated ASCs obtained from healthy donors were cultured in the presence of ascorbic acid (250 uM) and fibroblast growth factor-2 (1 ng/ml). Following 14 days of induction in adipogenic or osteogenic medium, adipogenic phenotype of ASCs was characterized by formation of cytoplasmic lipid droplets which were red in color and identified by Oil Red O staining. The osteogenic phenotype of ASCs was indicated by formation of multiple red bone nodules when stained with Alizarin Red. In addition, all ASCs were analyzed for representative markers (CD73, CD90 and CD105) of mesenchymal stem cells via flow cytometry within 5 passages. A non-specific antibody of mice was used as isotype control. Micrographic imaging and flow cytometric analysis were performed using ASCs at day 21 post-thawing. (A) Adipogenic and osteogenic differentiation of ASCs. 200 x magnification. (B) Immunophenotypic characterization of the cultured ASCs by FACS.

ASCs from healthy controls appeared as typical monolayers of spindle-shaped fibroblast-like cells during *ex vivo* expansion (Fig. 1A) with similar proliferative rate based on cell counting during passaging the cells up to passage 5 (data not shown).

ASCs are able to differentiate into specific cells when cultured in adipogenic or osteogenic medium 5, 7. In order to examine the potential of isolated ASCs for adipogenesis or osteogenesis, ASCs were cultured with adipogenic or osteogenic medium for 14 days, followed by an Oil-Red O or Alizarin Red S staining assay. Oil-Red-O staining of ASCs, after culture in adipogenic medium for 14 days, revealed the presence of lipid droplets (Fig. 1A). Positive staining of Alizarin Red S confirmed osteogenic induction following culture in osteogenic media (Fig. 1A). However, adipose tissue, in addition to committed adipogenic, endothelial progenitor cells and pluripotent vascular progenitor cells, also contains ASCs in cell culture conditions. In order to identify adherent cells from adipose tissue as MSC, the adherent cells were analyzed for surface markers CD44, CD90, and CD105 via fluorescence activated cell sorter (FACS). In accordance with the proposed criteria for the definition of MSCs 26, CD44, CD90 and CD105 (positive markers of MSCs) were expressed in more than 98.5% of the ASCs (Fig. 1B). This result suggests that isolated cells from human adipose tissues are mesenchymal stem cells.

### Adipose-derived mesenchymal stem cells indirectly decrease cell growth and increase proteins of p53/p21 in Huh7 cells

Previously, we reported that ASCs cultured at high density (40,000 cells/cm^2^) expressed type I IFNs and TRAIL. Cell death was induced in MCF-7 breast cancer cells and H460 lung cancer cells, via either IFN-β 15 or TRAIL 14. Therefore, ASCs were single-cultured or co-cultured at high density (40,000 cells/cm^2^) in order to investigate role of ASC in growth of Huh7 cells in the current study. According to previous studies, we hypothesized that ASCs induce cell death in Huh7 hepatocellular carcinoma cells.

**Figure 2:**
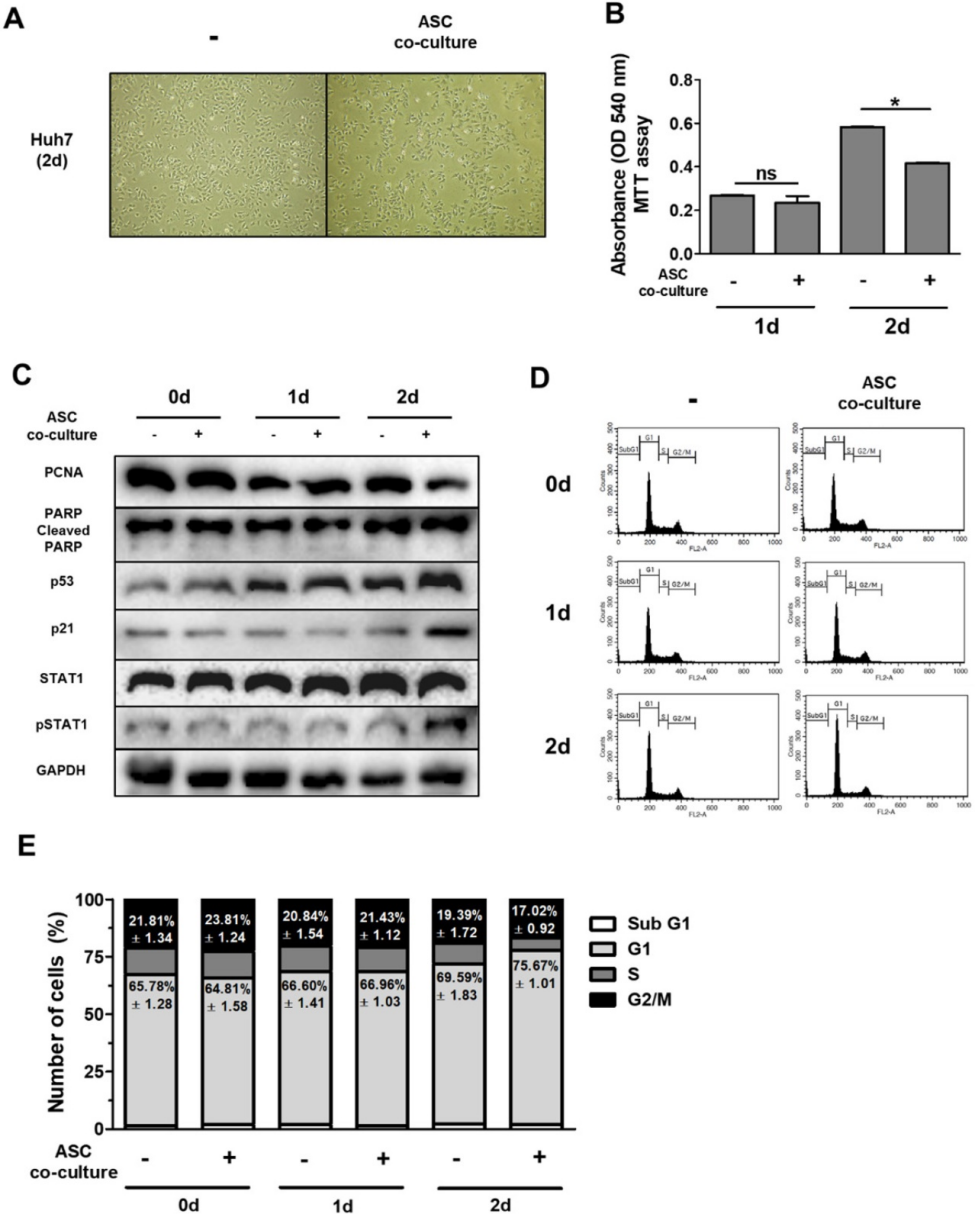
** Indirect co-culture with ASCs induces cell cycle arrest in Huh7 cells without apoptosis.** Huh7 cells were co-cultured with ASCs at doses of 40,000 cells/cm2 for 2 days. (A) Micrographs of light microscopy of Huh7 cells co-cultured with ASCs (left image) or not (right image) at day 2. 40 x magnification. (B) MTT assay showing cell viability of Huh7 cells co-cultured with ASCs or not at day 1 and day 2. (C) Western blot analysis indicating relative amount of PCNA, PARP, p53, p21, STAT1, p-STAT1 and GAPDH in lysates of Huh7 cells co-cultured with ASCs at day 1 and day 2. (D) FACS analyses of DNA content in Huh7 cells co-cultured with ASCs at day 1 and day 2. (E) Cell cycle distribution of Huh7 cells co-cultured with ASCs at day 1 and day 2. Error bars represent the mean ± SD from triplicate analyses. Data were obtained from one of three independent experiments. *P < 0.05. ns, no statistical significance.

In order to test this hypothesis, we indirectly co-cultured Huh7 cells with ASCs using a transwell system for 0-2 days. The results of this experiment indicate that Huh7 cells co-cultured with ASCs showed decreased absorbance values as demonstrated by MTT assay with no alterations (Fig. 2B) This suggests that ASCs indirectly inhibit cell growth but not apoptosis in Huh7 cells. Previous data suggest that increased p53 or p21 suppresses cell proliferation, thereby inducing cell cycle arrest 27-28. To further identify the mechanistic link in respect of IFN-β or TRAIL, the protein levels of genes for cell apoptosis (cleaved PARP), cell proliferation (PCNA), cell cycle (p21 and p53) and type 1 IFN signaling (pSTAT1) were measured in Huh7 cells by western blotting (Fig. 2C). In contrast to this, Huh7 cells co-cultured with ASCs showed decreased PCNA and increased p53/p21 and pSTAT1 at day 2 (Fig. 2C). Studies demonstrate that up-regulated p53/p21 positively correlates to cell cycle arrest in several cell types 28-29. To analyze the cell cycle in detail, Huh7 cells co-cultured with ASCs and analyzed by flow cytometry. There were no alterations in all populations of cell cycle between all groups at day 1 (Fig. 2D, E). However, the G1 population was upregulated and G2/M population was downregulated in Huh7 cells indirectly co-cultured with ASCs at day 2 (Fig. 2D, E). At day 2, it was also observed that Huh7 cells indirectly co-cultured with ASCs showed an increase in pSTAT1 (Fig. 2C).

### IFN-β, but not TRAIL, decreases cell growth and increases proteins of p53/p21 in Huh7 cells

**Figure 3:**
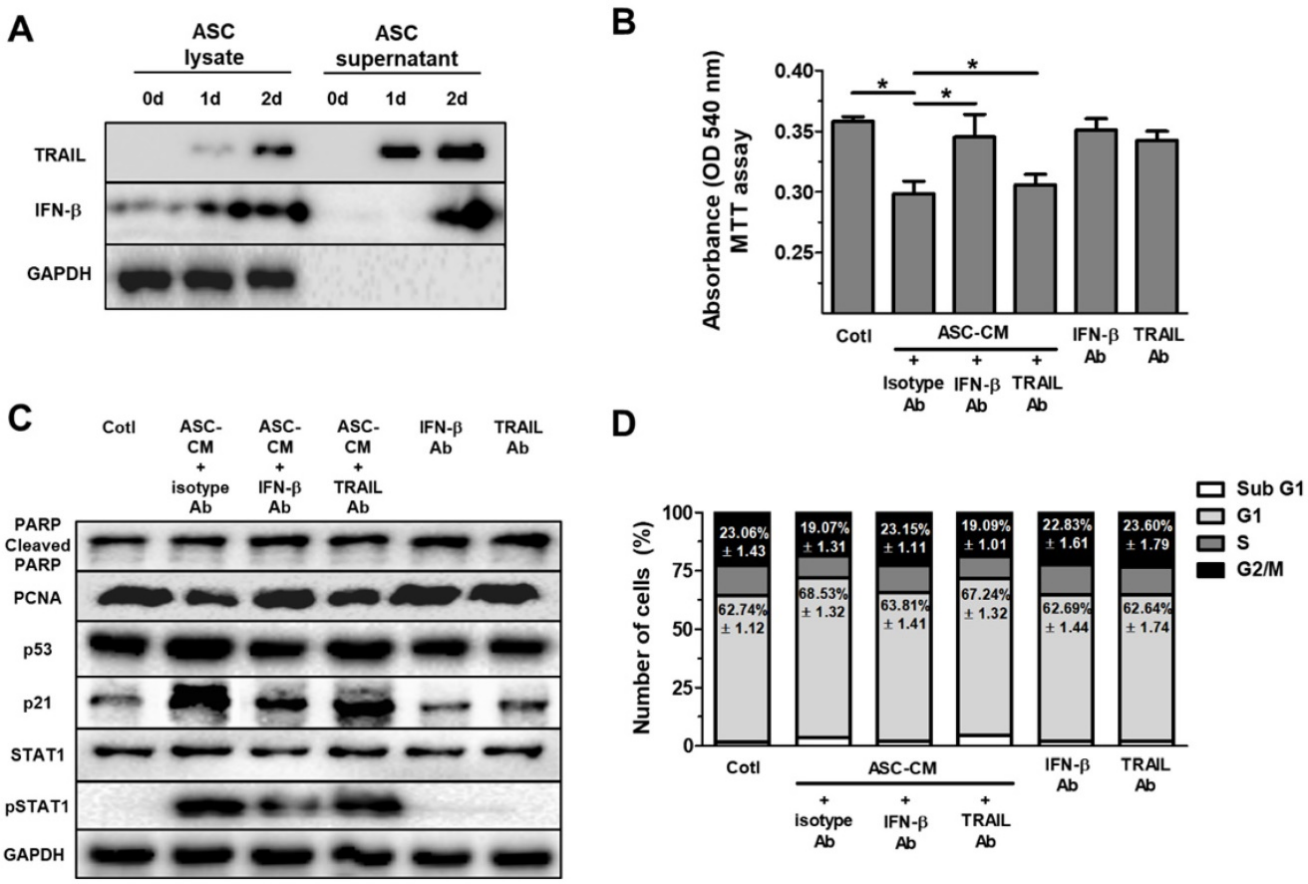
ASCs-secrete IFN-β, but not TRAIL, which induces cell cycle arrest in Huh7 cells through elevated p53/p21 in Huh7 cells. Huh7 cells treated with conditioned medium (CM) of ASCs after the neutralization of IFN-β or TRAIL. Huh7 cells were incubated with ASC-CM and treated with anti-IFN-β and/or anti-TRAIL neutralizing antibodies for 24 h. (A) Western blot analysis of cell lysate and supernatant in ASCs for IFN-β and TRAIL. GAPDH was used as cellular marker in cell lysate and supernatant. (B) MTT assay showing cell viability of Huh7 cells co-cultured with or without ASCs. (C) Western blot analysis showing relative amount of PCNA, PARP, p53, p21, STAT1, p-STAT1 and GAPDH in lysates of ASC-CM-treated Huh7 cells given anti-IFN-β or anti-TRAIL neutralizing antibodies, respectively. (D) Cell cycle distribution of ASC-CM-treated Huh7 cells given anti-IFN-β or anti-TRAIL neutralizing antibody, respectively. Error bars represent the mean ± SD from triplicate analyses. Data were obtained from one of three independent experiments. (For interpretation of the references to color in this figure legend, the reader is referred to the Web version of this article.) *P < 0.05. ns, no statistical significance.

Type 1 IFN activates JAK/STAT1 signaling *in vitro* 30. Additionally, our previous study revealed that IFN-β secreted by ASCs mediates cell cytotoxicity through JAK/STAT1 pathway in breast cancer cells 15. Therefore, these results prompted us to develop the hypothesis that ASCs indirectly suppress cell growth and increase p53/p21 in Huh7 cells via IFN-β. Before treatment of ASC-CM with Huh7 cells, lysates of ASC and supernatant of ASC were examined for IFN-β and TRAIL via western blot, separately. Western blot analysis indicated elevated IFN-β in lysates and supernatant of ASC for 0 to 2 day (Fig. 3D). In contrast, TRAIL was detected in the lysates and supernatant of ASCs during 1 to 2 day (Fig. 3A), supporting the hypothesis that ASC mainly suppress growth of Huh7 cells via IFN-β or TRAIL. To test the hypothesis, 2-day ASC-conditioned medium (ASC-CM) were concentrated, and used as an indirect co-culture media of ASCs for 2 days, which decreased cell viability in Huh7 cells.

Huh7 cells treated with ASC-CM and isotype control antibody showed decreased cell viability compared to Huh7 cells given isotype control antibody alone (data not shown). To evaluate the contribution of IFN-β to ASC-CM-induced decrease in Huh7 cell viability, we conducted experiments with IFN-β or TRAIL-neutralizing antibodies. Huh7 cells were pretreated with an isotype control antibody (0.2 mg/ml), IFN-β-neutralizing antibody (0.2 mg/ml), or TRAIL-neutralizing antibody (0.2 mg/ml) for 30 min prior to ASC-CM treatment. Blockade of IFN-β, but not TRAIL, inhibited ASC-CM-mediated suppression of cell viability 24 hours after co-treatment of the neutralizing antibody and ASC-CM (Fig. 3B), emphasizing the contribution of IFN-β in ASC-mediated growth inhibition and suggesting a minor role of TRAIL in reduced cell viability in Huh7 cells. In contrast, TRAIL blockade did not modify ASC-CM-induced growth inhibition in Huh7 cells (Fig. 3B). Furthermore, blockade of IFN-β, but not TRAIL, reduced down-regulation of PCNA and pSTAT1 and up-regulation of p53 and p21 in Huh7 cells treated with ASC-CM compared to isotype control at day 1 (Fig. 3C). Cell cycle analysis by FACS demonstrated that the G1 population was reduced and the G2/M population was increased in ASC-CM-treated Huh7 cells given an IFN-β neutralizing antibody (Fig. 3D), compared to Huh7 cells cultured with ASC-CM and an isotype control antibody. Whilst there were no changes in all populations of cell cycle between ASC-CM-treated Huh7 cells given isotype control antibody and ASC-CM-treated Huh7 cells given a neutralizing antibody to TRAIL (Fig. 3D). This result provides a direct example that IFN-β/STAT1 signaling contributes to ASC-induced cell cycle arrest in proliferation of Huh7 cells.

### IFN-β decreases cell growth in Huh7 cells through the JAK/STAT1 signaling pathway

**Figure 4:**
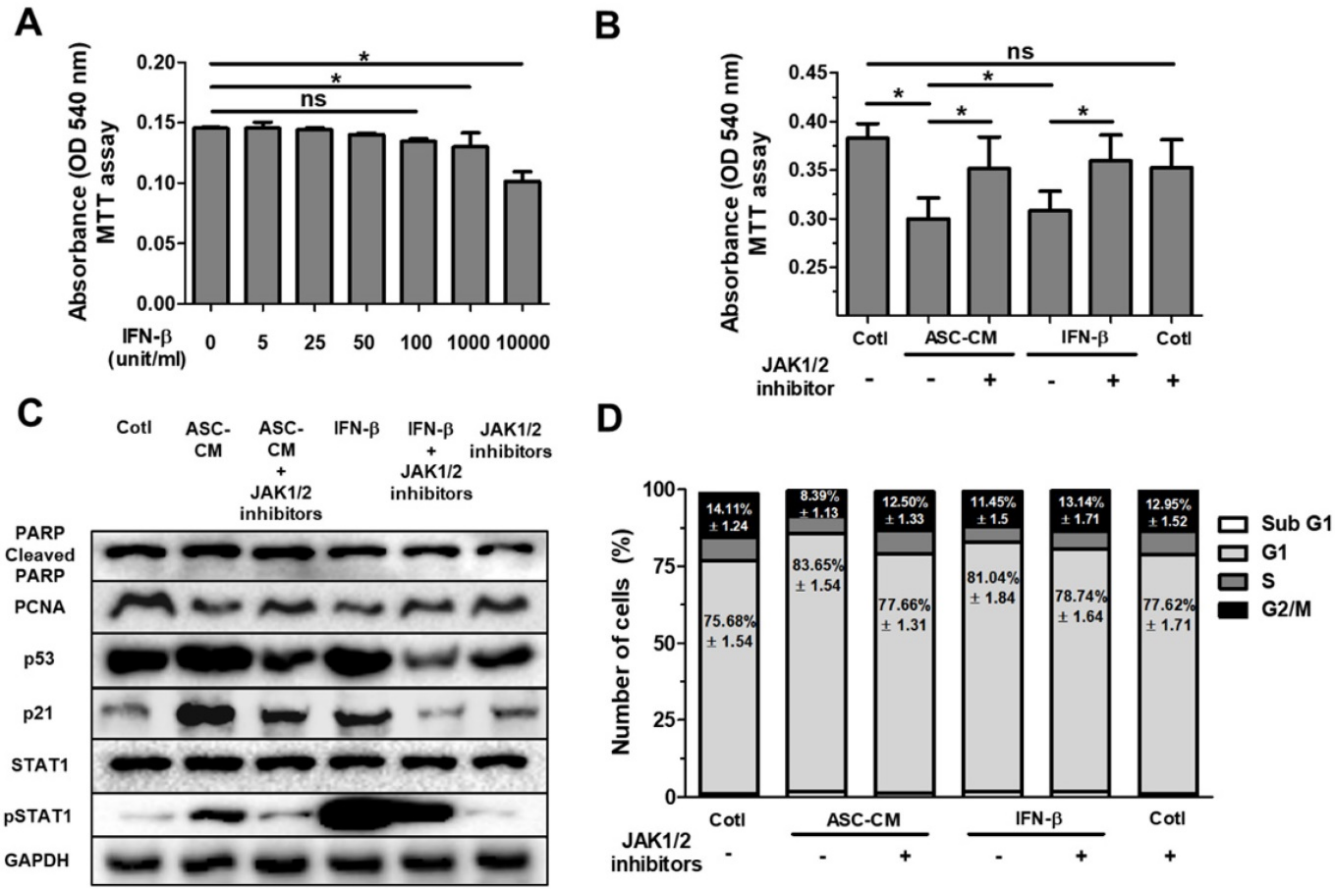
** ASC-secreted IFN-β induces cell cycle arrest in Huh7 cells via the JAK/STAT1 pathway.** Huh7 cells were treated with ASC-CM or IFN-β, and treated with JAK1 (25 nM) or JAK2 (25 uM) inhibitors for 24 h. (A) MTT assay showing cell viability of Huh7 cells treated with IFN-β (5 unit/ml to 10000 unit/ml) in a dose-dependent manner. (B) MTT assay showing cell viability of Huh7 cells given ASC-CM or IFN-β (1000 unit/ml) in the presence of JAK1 (25 nM) or JAK2 (25 uM) inhibitors. (C) Western blot analysis showing relative amount of PCNA, PARP, p53, p21, STAT1, p-STAT1 and GAPDH in lysates of ASC-CM or IFN-β (1000 unit/ml) treated Huh7 cells given JAK1 (25 nM)/JAK2 (25 uM) inhibitors or not. (D) Cell cycle distribution of ASC-CM or IFN-β (1000 unit/ml) treated Huh7 cells given JAK1 (25 nM)/JAK2 (25 uM) inhibitors or not. Error bars represent the mean ± SD from triplicate analyses. Data were obtained from one of three independent experiments. *P < 0.05. ns, no statistical significance.

We previously reported that IFN-β stimulates cytotoxicity through the JAK/STAT1 pathway in MCF-7 human breast cancer cells 15. In order to determine optimal concentration of IFN-β treatment, Huh7 cells were treated with IFN-β (5 unit/ml to 10000 unit/ml) for 24 hours in a dose-dependent manner, followed by MTT assay for cell viability (Fig. 4A). Analysis of MTT assay showed that a dose of IFN-β (1000 unit/ml) indicated a similar decrease in cell viability (Fig. 4A) compared with Huh7 cells treated with ASC-CM alone (Fig. 4B). Based on this result (Fig. 4A), to investigate the impact of JAK/STAT1 pathway on IFN-β-induced inhibitory effects on cell growth, Huh7 cells were treated with ASC-CM or IFN-β (1000 unit/ml) and/or JAK1 (25 nM)/JAK2 (25 μM) inhibitors.

Western blot analysis indicated that Huh7 cells treated with ASC-CM or IFN-β increased p53, p21 and pSTAT1, and decreased PCNA (Fig. 4C), suggesting that IFN-β alone induces upregulation of p53 and p21 with activated STAT1 in Huh7 cells. Blockade of JAK/STAT1 pathway by co-treatment of JAK1/JAK2 inhibitors suppressed ASC-CM or IFN-β-induced decrease in cell viability (Fig. 4B). Consistent with these results, Western blot analysis revealed that PCNA, p53, p21 and pSTAT1 were decreased in ASC-CM and IFN-β-treated Huh7 cells given JAK1/JAK2 inhibitors compared to Huh7 cells given ASC-CM or IFN-β alone (Fig. 4C) This emphasizes the contribution of the IFN-β→JAK/STAT1 pathway in ASC-CM-induced suppression of growth of Huh7 cells. Having observed that IFN-β treatment decreased cell viability through the JAK/STAT1 pathway in Huh7 cells, we further analyzed cell cycle populations via flow cytometry in order to confirm decreased cell growth in detail. Results indicate that the G1 population increased and G2/M population decreased in ASC-CM or IFN-β-treated Huh7 cells compared to JAK1/JAK2 inhibitor-treated Huh7 cells given ASC-CM or IFN-β (Fig. 4D). This result is consistent with data for decreased PCNA in Huh7 cells treated with IFN-β or ASC-CM (Fig. 4C). There were no significant alterations in other populations of cell cycle between all groups (Fig. 4D). These data collectively demonstrate that ASC-induced growth inhibition is directly mediated by IFN-β through the JAK/STAT1 pathway in Huh7 cells.

### IFN-β does not affect resistance of Huh7 cells to TRAIL resistance

**Figure 5:**
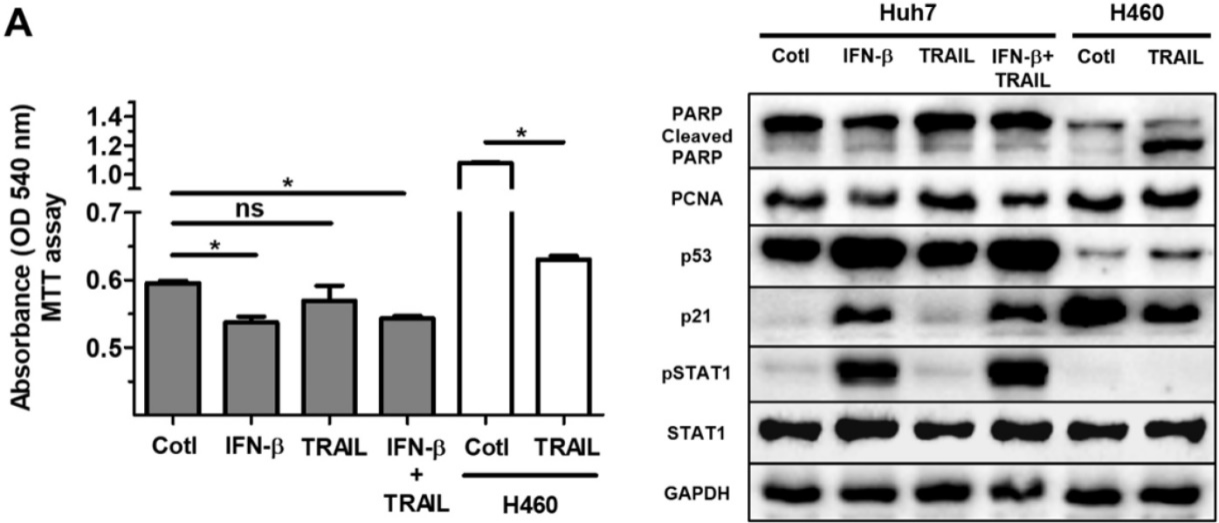
** Treatment of IFN-β does not overcome resistance of TRAIL in Huh7 cells.** Huh7 cells were treated with IFN-β (1000 unit/ml) and/or TRAIL (100 ng/ml) for 24 h. H460 cells were used as TRAIL-sensitive lung cancer cells to confirm TRAIL activity. Both Huh7 and H460 cells were seeded as a density of 1 x 10^5^ /wells in 6 well plates, followed by treatment of IFN-β and/or TRAIL for 24 h. (A) MTT assay showing cell viability of Huh7 cells and H460 cells given IFN-β (1000 unit/ml) and/or TRAIL (100 ng/ml). (B) Western blot analysis showing relative amount of PCNA, PARP, p53, p21, STAT1, p-STAT1 and GAPDH in lysates of Huh7 cells given IFN-β (1000 unit/ml) and/or TRAIL (100 ng/ml). Error bars represent the mean ± SD from triplicate analyses. Data were obtained from one of three independent experiments. *P < 0.05. ns, no statistical significance.

Many primary cancer cells including Huh7 cells are resistant to TRAIL treatment 31. Overcoming the intrinsic or acquired TRAIL resistance of hepatic cancer is desirable for TRAIL-mediated cancer therapy. Whilst we did not detect TRAIL in the lysate or supernatant of ASC in the current study (Fig. 3A), only small protein volumes can be studied by western blot analysis. In addition, a small amount of TRAIL may impact IFN-β-induced effects on cell growth and proliferation in Huh7 cells. Having observed the IFN-β-mediated decrease in Huh7 cell viability, we next examined whether treatment of IFN-β modifies TRAIL resistance of Huh7 cells. Results of experiments showed that Huh7 cells given IFN-β (1000 unit/ml) and TRAIL (100 ng/ml) show comparable cell viability compared with Huh7 cells treated with IFN-β or TRAIL, suggesting that IFN-β might be insufficient to overcome TRAIL resistance. Western blot analysis also showed no alterations in cleaved PARP, PCNA, p53 and p21 between Huh7 cells co-treated with IFN-β and TRAIL and Huh7 cells given IFN-β or TRAIL alone (Fig. 5B). Additionally, TRAIL-sensitive H460 human lung cancer was used as TRAIL-responsive cancer cells in order to evaluate activity and concentration of TRAIL, and we confirmed activity and concentration (100 ng/ml) of TRAIL is effective and sufficient to induce apoptosis in H460 cells with apoptotic morphology, decreased cell viability and increased cleaved PARP (Fig. 5A,B). Additionally, Huh7 cells were indirectly co-cultured with ASCs during 5 days, as secreted TRAIL by ASCs is high after 2 days of ASC culture, followed by MTT assay in Huh7 cells. However, Huh7 cells co-cultured with ASCs indicated decreased cell viability rather than increased apoptosis (data not shown). This result confirms that TRAIL by ASCs does not induce apoptosis in Huh7 cells with or without IFN-β.

## Discussion

Herein, we showed that ASC-secreted IFN-β was sufficient to inhibit growth of cells as assessed via cell viability, increased p53/p21 and G1 population. Conversely, TRAIL failed to demonstrate any cytotoxic effects at high concentration (100 ng/mg) due to TRAIL resistance of Huh7 cells, indicating that IFN-β may be a superior alternative to TRAIL as an anti-cancer molecule in TRAIL-resistant cancer cells. However, intravenous IFN-β administration may have side effects in major organs via affecting non-tumor cells and the therapeutic effect of IFN-β may be diminished due to the short half-life time in the body. Since ASCs have the property to migrate to tumor mass 32, the migration of ASCs secreting IFN-β might be specifically effective to target proliferation of hepatocellular carcinoma cell in tissue.

Notwithstanding the advantages of ASCs as a potential agent to inhibit hepatocellular carcinoma, ASCs are not without limitations. For example, ASCs have the potential to develop into tumors, which may inhibit ASC-based clinical trials in cancer patients. Another limitation of ASCs is their susceptibility to phagocytosis by neutrophils and macrophages *in vivo* 33. This may be difficult to overcome in a tumor environment infiltrated with several immune cells including the neutrophil and macrophage. Yet, in the context of a tumor microenvironment with few immune cells, localization of ASCs to the tumor may exhibit effective anti-cancer activity. In contrast, ASCs may suppress dysregulated inflammation-promoted tumorigenesis through paracrine effects as ASCs secrete PGE2 34-35, an immune-suppressive molecule. Following environmental stress-induced DNA damage, many cancer cells show sustained arrest in the G2 phase of the cell cycle 36. It is reported that this arrest can only be sustained p53 is present in the cell and capable of transcriptionally activating the cyclin-dependent kinase inhibitor p21 37. After disruption of either the p53 or the p21 gene, γ-radiated colorectal cancer cells progress into mitosis and exhibit a G2 arrest due to failure of cytokinesis 28-29, p53 and p21 appear to be essential for maintaining the G2 checkpoint in human cells. However, in this study, we found that IFN-β treatment resulted in upregulation of G1 arrest, suggesting that IFN-β treatment may decrease cellular proliferation without DNA damage in Huh7 cells. DNA damage leads to phosphorylation of p53 at Ser15 and Ser20, resulting in reduced interaction between p53 and its negative regulator, the oncoprotein MDM2 38. MDM2 suppresses p53 accumulation by targeting it for ubiquitination and proteasomal degradation 38. Our study did not investigate the subtype of phosphorylated p53 and MDM2-mediated suppression on p53 levels in Huh7 cells given IFN-β. Additional studies are necessary to investigate a mechanistic link between phosphorylated p53, MDM2 and cell cycle arrest in Huh7 cells treated with IFN-β. Interestingly, Serhal et al recently conducted a similar experiment, wherein inhibitory effect of adipose derived-mesenchymal stem cells (ADMSCs) on HepG2 and PLC-PRF-5 hepatocellular carcinoma cells 24. The mechanistic explanation for this observation is unclear, but the data suggest that the both HepG2 and PLC-PRF-5 cells show apoptosis with increased p53 and Rb proteins. However, the association of JAK/STAT pathway in ASC-induced p53/Rb activation in the two cells has not been investigated.

Contrary to our expectations, Huh7 cells co-cultured with ASC did not induce apoptosis, but did induce cell cycle arrest via the IFN-β→JAK/STAT1 pathway. We expected that TRAIL by ASC would acts as an apoptosis-inducing factor in lieu of IFN-β, as TRAIL was detectable in the lysates and supernatant of ASCs. The fact that IFN-β inhibited cell proliferation of Huh7 cells via cell cycle arrest, but did not increase TRAIL sensitivity in the Huh7 cells, suggests that IFN-β might induce synergy with anti-cancer agents responsive to TRAIL-resistant cells.

IFN-β reportedly enhances the production of hepatocyte growth factor (HGF) from MSCs via STAT1-3-dependent signaling 39. HGF is the only known high-affinity cytokine for the c-Met receptor. Binding of HGF to c-Met causes receptor multimerization-mediated phosphorylation and results in signaling cascades, thereby inducing the phosphorylation of multiple downstream effectors including RAS/MAPK and PI3K/AKT 40. HGF/c-Met signaling is reportedly essential for promoting cell growth and survival in hepatic cancer cells 41. Herein, IFN-β independently decreased the growth of Huh7 cells irrespective of TRAIL. However, treatment of HCC model mice with IFN-β-secreting MSCs might not reflect any of the changes in tumorigenesis owing to IFN-β-induced HGF production. Furthermore, targeting HGF/c-Met signaling might result in side effects during the treatment of HCC patients, since HGF/c-Met signaling is responsible for the defensive response to hepatic damage 42. Recent studies report the modulation of miR-221/222 as a therapeutic strategy to counteract their TRAIL resistance. A miRNA sponge for miR-221 has been developed and shows strong suppression of this miRNA together with upregulation of its targets within HCC cells 43. Significant efforts have also been made to exploit the innate ability of MSCs to traffic to sites of inflammation, including those present in cancer, to deliver a variety of therapeutic interventions 44-45. Here we provide a possible strategy by drug carrier of ASCs secreting IFN-β/TRAIL plus anti-miR-221/222, to suppress TRAIL resistance. Taken together, our study provides evidence that ASCs can be used to inhibit hepatocellular carcinoma, potentially by the IFN-β-induced JAK/STAT1 pathway. Though the ability of ASCs to suppress cell growth may not be surprising, our study highlights the IFN-β-induced mechanistic link between JAK/STAT1 and p53/p21. Further studies are critical to evaluate the extent to which the suppressive effects of ASCs or IFN-β on cell growth may be used *in vivo*.

## Material and Methods

### Cell culture

ASCs were isolated by collagenase digestion of lipoaspirates obtained from three healthy donors undergoing liposuction and who had given their informed consent. All protocols were approved by the Institutional Review Board of Yonsei University Wonju College of Medicine (CR319098) and Institutional Biosafety Committee (19-6 and 19-7) of Yonsei University Wonju College of Medicine.

Briefly, as previously described 24, 46, lipoaspirates were washed extensively with Hank's Balanced Salt Solution (HBSS) (Life Technologies, Carlsbad, CA, USA) to remove debris, before treating with collagenase (0.025%) in HBSS for an hour at 37 °C under gentle agitation. Debris of adipose tissues was eliminated by filtration with a 100-μm mesh filter (Cell Strainer, Becton Dickinson, Franklin Lakes, NJ, USA). 5 × 10^6^ mononuclear cells were seeded in 100-mm culture plates with DMEM with 10% FBS and penicillin/streptomycin. After 2 days, the culture medium of mononuclear cells was changed to remove non-adherent cells. Thereafter, the cell culture medium was changed twice weekly, and cells were passaged with 0.25% trypsin/0.1% EDTA (Life Technologies, Carlsbad, CA, USA) until reaching 80% confluency. For experiments, cells were seeded with the indicated cell dose (40,000 cells/cm^2^; hereafter referred to as 40 K) and cultured for the indicated times. Conditioned medium (CM) was collected, filtered (with a 0.45 μm filter), and stored at -80 °C until needed. The ASCs were incubated at 37 °C and 5% CO_2_ and their medium was replaced every 3-4 days. When the cells reached 70%-80% confluence MSCs were trypsinized and passaged. Indirect co-culture of Huh7 cells with ASCs was performed using a transwell plate (Corning, New York, USA). ASCs were cultured at 40 K (upper chamber) and then co-cultured with Huh7 cells (lower chamber) for 2 days in order to observe cell morphology, sample protein lysate and analyze cell cycles.

### Adipogenic and osteogenic differentiation of ASCs

ASCs (passage 4) were cultured in a medium that contained either adipogenic (isobutylmethylxanthine [0.5 μM], dexamethasone [1 μM], insulin [10 μM], and indomethacin [200 μM]) or osteogenic (dexamethasone [0.1 μM], β-glycerophosphate [10 μM], and ascorbate-phosphate [50 μM]) reagents. After 14 days of treatment, osteogenic differentiation was evaluated by the analysis of cytoplasmic calcium with Alizarin Red S kit and adipogenic differentiation was analyzed through cytoplasmic lipid accumulation, which was visualized using Oil-Red-O staining by a light microscope (Nikon Corporation, Tokyo, Japan). All reagents and materials for adipogenic and osteogenic differentiation were purchased from Sigma-Aldrich. ASCs were cultured in the regular medium as undifferentiated control.

### *In vitro* ASC characterization

Established ASCs from both healthy donors were examined for cellar morphology during passage, and characterized for CD markers characterized with mesenchymal stem cell via flow cytometry. The primary antibodies for analysis were anti-human CD73 (BD Biosciences, San Jose, CA, USA), anti-human CD90 (BD Biosciences, San Jose, CA, USA) and anti-human CD105 (BD Biosciences, San Jose, CA, USA) which were used at the manufacturer's recommendations.

### Inhibitors and other treatments

Huh7 cells were washed once with PBS before treatment with ASC-CM, IFN-β (8499-IF; R&D system, Minneapolis, MN, USA) and TRAIL (375-TL; R&D system, Minneapolis, MN, USA) in DMEM with 10% FBS. Huh7 cells were co-treated with the JAK1 inhibitor (S8765; Selleck Chemicals, Houston, TX, USA) and JAK2 inhibitor (SD-1029; Santa Cruz biotechnology, Dallas, TX, USA) for 30 min before the addition of ASC-CM, IFN-β and TRAIL then continuously during the experiment unless otherwise described. Isotype control antibody (1-001-A; R&D system, Minneapolis, MN, USA) and individual antibody to neutralize IFN-β (MAB814; R&D system, Minneapolis, MN, USA) or TRAIL (AF375; R&D system, Minneapolis, MN, USA) was also added to supernatant of DMEM with 10% FBS for 30 min before treatment of ASC-CM, IFN-β and TRAIL.

### MTT assay

Huh7 cells were seeded at 1 × 10^4^ cells/cm^2^ in 6 well plates and cultured for 1 day. As the CM of ASCs may be deficient in glucose and amino acids 14, this CM (ASC-CM) obtained from ASC may be possible to decrease cell viability of Huh7 cells due to depleted nutrients. In order to prevent this possibility, the CM from a 3-day culture of ASCs was concentrated using Amicon® Ultra-15 Centrifugal Filter Units (C7715; Millipore, Darmstadt, Germany). Methylthiazolyldiphenyl-tetrazolium bromide (MTT; Sigma-Aldrich) dissolved in PBS was added to each well (10 mg/ml) and incubated at 37 °C for an hour. MTT formazan was dissolved in 200 μL dimethyl sulfoxide and incubated for 15 min. After incubation, absorbance of each well was measured at 570 nm using a microplate reader (BioTek Instruments, Winooski, VT, USA).

### Immunoblot analysis

For western blot analyses, proteins of Huh7 cells were probed using the primary antibodies as indicated then horseradish peroxidase-coupled secondary antibodies at 1:3000 (Cell signaling technology) after 10% SDS-PAGE separation and nitrocellulose membrane transfer 47. The proteins were transferred to PVDF membrane, blocked with 5% non-fat dry milk and probed for PARP (#9532; Cell Signaling Technology), PCNA, (sc-56; Cell Signaling Technology), p53 (sc-126; Cell Signaling Technology), p21 (sc-6246; Cell Signaling Technology), STAT1 (#9172; Cell Signaling Technology), P-STAT1 (#9171; Cell Signaling Technology) and GAPDH (#2118; Cell Signaling Technology). GAPDH was used as a loading control. Immunoblots were developed using ECL chemiluminescent reagent (Bio-Rad Laboratories, San Francisco, CA, USA). Immunoreactive proteins were detected using Super Signal West Pico Chemiluminescent Substrate (Pierce, Rockford, IL) and quantified by densitometry using ChemiDoc XRS+ System (Bio-Rad Laboratories, San Francisco, CA, USA). To detect molecules of cellular signaling in Huh7 cells, lysate of Huh7 cells were precipitated with 10% trichloroacetic acid followed by western blot using the primary antibodies. Cellular proteins were extracted with sample buffer (300 mM Tris-HCl, 10% SDS, 20 mM EDTA, 25% ß-mercaptoethanol, 0.1% bromophenol blue and 50% glycerol) for 10 min at 4°C. After heat inactivation of cellular proteinases at 100 °C, the proteins of cell lysates were analyzed by western blot as described.

### Flow cytometry

Huh7 cells were harvested by trypsinization and resuspended in DMEM supplemented with 10% fetal bovine serum (FBS) in order to neutralize trypsin. After neutralization by media with 10% FBS, Huh7 cells were centrifuged, and the pellet of Huh7 cells was washed twice with PBS, and the cell were resuspended by a vortex mixer. Thereafter, the tube of Huh7 cells were washed with 70% ice-cold ethanol for 30 sec. The suspensions of Huh7 cells were centrifuged at 400 x g for 5 min at room temperature. After decanting all the supernatant, Huh7 cells was washed twice with PBS, followed by incubation with RNase for 30 min. The population of Huh7 cells was then split, and cells were stained with propidium iodide (BD Biosciences, San Jose, CA, USA) for 30 min at 4 ℃. Huh7 cells stained with propidium iodide were washed with PBS through centrifugation (400 x g, 5 min) to decrease the cellular debris or non-specific fluorescent signal by unstained propidium iodide. Huh7 cells were analyzed by FACScalibur (BD Biosciences, San Jose, CA, USA) with BD CellQuest Pro Software (BD Biosciences, San Jose, CA, USA). Individual solutions containing trypsin, trypsin-neutralizing reagent or propidium iodide, respectively, were obtained from BD Cycletest™ Plus DNA Kit (BD Biosciences, San Jose, CA, USA).

### Statistical analysis

All *in vitro* experiments were performed in triplicate and the results expressed as mean ± SEM. Data presented were representative of three independent experiments. P values were obtained using a paired 2-tailed Student's t test (Mann-Whitney U test). All statistical analyses were performed with GraphPad Prism 6.0 software (GraphPad Inc., La Jolla, CA). p< 0.05 was considered statistically significant and denoted as *p< 0.05 and **p< 0.01 ***p< 0.001.
